# Integrated Computational Tools for Identification of CCR5 Antagonists as Potential HIV-1 Entry Inhibitors: Homology Modeling, Virtual Screening, Molecular Dynamics Simulations and 3D QSAR Analysis

**DOI:** 10.3390/molecules19045243

**Published:** 2014-04-23

**Authors:** Suri Moonsamy, Radha Charan Dash, Mahmoud E. S. Soliman

**Affiliations:** School of Health Sciences, University of KwaZulu-Natal, Westville, Durban 4001, South Africa

**Keywords:** CCR5 antagonists, HIV-1 entry inhibitors, homology modeling, virtual screening, molecular dynamic simulations, 3D QSAR analysis, computer-aided drug design

## Abstract

Using integrated *in-silico* computational techniques, including homology modeling, structure-based and pharmacophore-based virtual screening, molecular dynamic simulations, per-residue energy decomposition analysis and atom-based 3D-QSAR analysis, we proposed ten novel compounds as potential CCR5-dependent HIV-1 entry inhibitors. Via validated docking calculations, binding free energies revealed that novel leads demonstrated better binding affinities with CCR5 compared to maraviroc, an FDA-approved HIV-1 entry inhibitor and in clinical use. Per-residue interaction energy decomposition analysis on the averaged MD structure showed that hydrophobic active residues Trp86, Tyr89 and Tyr108 contributed the most to inhibitor binding. The validated 3D-QSAR model showed a high cross-validated *r*_cv_^2^ value of 0.84 using three principal components and non-cross-validated *r*^2^ value of 0.941. It was also revealed that almost all compounds in the test set and training set yielded a good predicted value. Information gained from this study could shed light on the activity of a new series of lead compounds as potential HIV entry inhibitors and serve as a powerful tool in the drug design and development machinery.

## 1. Introduction

The Human Immunodeficiency Virus type 1 (HIV-1) infection, the causative agent of Acquired Immunodeficiency Syndrome (AIDS) [1], still remains a fatal human health-threatening disease [2]. An estimated 34 million people live with HIV/AIDS worldwide [3,4]. The overall global estimate is that approximately 22.9 million of these individuals live in sub-Saharan Africa [3,4].

In AIDS therapy, the fundamental strategy is to inhibit viral replication and hence, to slow down the destruction of the immune system and prolong the lives of infected individuals. Currently, a number of viral targets are being used to develop anti-HIV drugs; which are essential for viral replication and survival, and these include, protease enzyme (PR) [5], reverse transcriptase (RT) [6] and integrase (IN) [7]. Several drugs which are currently in clinical use have been developed to inhibit these potential viral targets, and such as integrase inhibitors, reverse transcriptase inhibitors and protease inhibitors [8].

Numerous concerns regarding the long-term side effects of antiretroviral drugs and the increasing transmission of resistant variants accentuates the requirement to identify new classes of drugs, which are able to efficiently suppress HIV-1 replication [2]. Therefore, there is an on-going need for novel therapeutics, which can prevent the entry of HIV-1 into its target cells [9,10].

The entry of the HIV virus into its target cell is mediated by the specific interactions of the target cell itself, such as the interaction between gp120 viral envelope glycoprotein and the plasmatic membrane receptors [11]. In turn, these specific interactions produce conformational alterations in both the glycoprotein and in the membrane receptors that facilitates fusion of the HIV virus and the target cell. Numerous studies have evaluated the role of CD4 and its interaction with gp120 and concluded that the CD4-gp120 interaction is a crucial component, but is not adequate for the disease to become established [12,13].

Besides CD4, recently certain chemokine receptors (CCRs) belonging to the G-protein coupled receptor superfamily (GPCRs) have been identified as co-targets crucial for viral entry into target cells [9,14]. Different CCRs and counterpart chemokine ligands (RANTES, MIP-1alpha and MCP2) are responsible for signaling regulation within immune cells and therefore are potential target systems for preventing virus-cell fusion. Several studies have reported on the identification and characterization of diverse CCRs [15]. Besides a single CCR that is viral strain-dependent, the majority of CCR strains are R5 isolates, which are transmitted during sexual intercourse and act on CCR5 throughout the disease [15].

CCR5 has proven to be an important pharmaceutical target in the contexts of HIV-1 and other inflammatory diseases. This chemokine receptor functions as an integral protein in HIV-1 entry into host cells by acting as a crucial co-receptor for the gp120 viral envelope glycoprotein [1]. Furthermore, experimental data revealed the importance of CCR5 in HIV-1 transmission. It was reported that individuals that are homozygous for the 32-base pair deletion for the CCR5 allele produce a defective CCR5 co-receptor and are resistant to R5-tropic HIV-1 infection, however are otherwise generally healthy [16]. This fact has been an instigating factor in the past decade for identifying anti-HIV agents that specifically targets CCR5-mediated entry mechanism. Furthermore, this implies that functional inhibition of CCR5 may help protect against infection without provoking damage to patients. Thus, blocking viral entry using small-molecule antagonists selective for CCR5 might provide a new and more effective type of anti-HIV drug.

Although, the concept of designing small molecule CCR5 antagonists has been investigated before [1,10,17]; to date, no structural information about the precise binding site of CCR5 with any FDA-approved inhibitor is available. Several studies have reported CCR5 modeling of “potential” hit leads using computational approaches, including virtual screening, molecular docking, molecular dynamic stimulations and pharmacophore modeling. Perez-Nueno *et al.*, reported a detailed comparative report of ligand-based and receptor-based virtual screening methods to unveil potential HIV entry inhibitors for CXCR4 and CCR5 receptors [18]. It has been documented that structure-based virtual screening methods yield better results as compared to ligand-based approaches. Afantitis *et al.* and Aher *et al.* identified CCR5 antagonists derived from 1-(3,3-diphenylpropyl)-piperidinyl amides using virtual screening and quantitative structure-activity relationships (QSAR) studies [19,20]. In a previous report by Kellenberger *et al.* structure-based techniques were used to model the physics of protein-ligand interactions in conjugation with combined 2D and 3D structure-based techniques [21]. Researchers have developed new approaches of combining computational molecular modeling methodologies, for example, molecular docking, 3D-QSAR, comparative receptor modeling and virtual screening to discover potential CCR5 HIV-1 entry inhibitor drugs [1]. Xu *et al.* studied the detailed interactive relationship between CCR5 and its inhibitors using a docking-based/ 3D-QSAR strategy along with protein modeling and MD stimulation [2]. However, in other mechanistic studies of protein-ligand entry inhibitor interactions, investigators have used homology modeling, molecular docking and molecular dynamic stimulation techniques [1].

**Figure 1:**
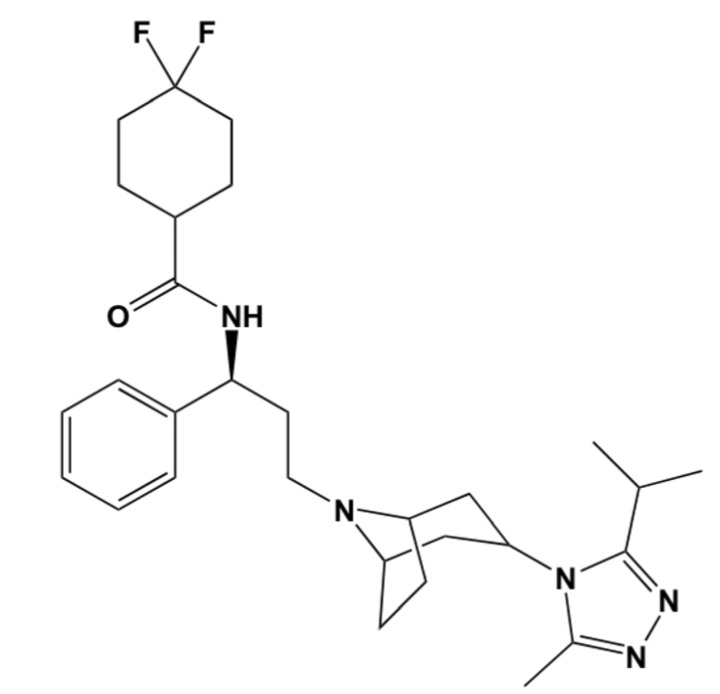
2D Structure of the known FDA-approved CCR5 antagonist maraviroc.

To this end, in this report, via hybrid structure-based and ligand-based virtual screening, we aim to identify novel CCR5 antagonists as potential HIV-1 entry inhibitors. A human CCR5 homology model template and maraviroc, a known FDA-approved CCR5 antagonist (Figure 1), were used as prototypes. Virtual screening of ligand-based compound libraries were generated via two distinct yet complimentary approaches: (a) structural similarity-based compound generated library—this library generated compounds that bear a 2D structural similarity to the reference drug maraviroc, whereas the (b) Pharmacophore-based generated library—this library generated compounds that contained the pharmacophoric features of the reference drug structure. Merging these independent compound libraries allowed us to ensure that our generated hit lead library encompassed structural units with diversity, yet with mutual pharmacophoric and structural features. Furthermore, docking calculations were computed using the generated ligand-based compound libraries against the CCR5 enzyme.

To validate our docking calculations, the same docking approach adopted for the ligand-based libraries was then performed on a set of compounds with known experimental data obtained from inhibition assays against HIV-1 CCR5 and these results were compared against experimental data. Since molecular docking may not be a true reflection of the stability of an ligand-enzyme complex, therefore, in order to gain more insight into the stability of the resulted docked systems, the nature of the overall interaction themes between the generated ligands and the target protein, and the specific amino acids involved in ligand binding, we performed 1 ns MD simulations followed by extensive post-dynamic analysis.

We took our study a step further by obtaining a set of 35 novel oxamino-piperidino-piperidine amide analogs with available IC_50_ (mM) data taken from literature for the development of our atom-based 3D-QSAR model [1].

It is worth mentioning that the three dimensional (3D) CCR5 structure is not yet available. However, homology-modeling of CCR5 has been performed before [9]. Therefore, in this study, the actual human CCR5 homology model was developed using the crystal structure of CXCR4 as a structural template. Information gained from this study could shed light on the activity of a new series of lead compounds as potential HIV entry inhibitors. This study should serve as a powerful tool in the drug design and development machinery.

## 2. Computational Methods

### 2.1. Homology Modeling of CCR5

In order for our molecular docking study to be executed, the crystal structure of human CCR5 was homology modeled using the human CCR5 protein sequence retrieved from the UniProt database [22] (Uniprot ID: P51681). The actual homology model of CCR5 was developed using the crystal structure of CXCR4 (PDB ID: 3ODU) as a structural template and using the Modeler software [23] add-on in Chimera [24]. Hydrogen atoms were included in our enzyme model, whilst all other important active site residues were identified using Chimera Multi-align Viewer [24].

### 2.2. Maraviroc Structure Acquisition and Preparation

Maraviroc, the known FDA-approved CCR5 antagonist, was obtained in a mol2 file format from the DrugBank [25,26,27]. This CCR5 antagonist had its geometry optimized and energy minimized using the MMFF94 force field found in Avogardros software [28]. Thereafter, for subsequent analyses, maraviroc was kept in the mol2 format.

### 2.3. Ligand Library Generation

#### 2.3.1. Structural Similarity-Based Compound Library Generation

Maraviroc was used as the template for generating the 2D shape similarity-based compounds library from the Zinc Database. The maraviroc structure was drawn using the MarvinSketch software [29]. This reference template was used uploaded and queried the Zinc Database for all structures that had greater than 60% shape similarity to maraviroc. The query search generated a total of 1,002 compound hits. As explained in our earlier report, certain physiochemical filters were implemented to enhance the structural query process [3]. In this study, the default physiochemical filter was set at drug-like qualities. Only compounds with molecular weight between 150 and 500 kDa were selected—this resulted in 480 hits. Other criteria were imposed to ensure the inclusion of the maximum number of compounds, such as the compounds had to have an xlog P between −4 and 5, a net charge between −5 and 5, rotatable bonds between 0 and 8, a polar surface area of between 0 and 150, have hydrogen bond donors/acceptors between 0 and 10, and polar desolvation between 0 and 1 kcal/mol whereas compounds must have an apolar desolvation between −100 and 40 kcal/mol. Thereafter, these compounds were downloaded as a single mol2 file format and were individually separated into mol2 files using the Molegro Molecular Viewer (MMV) software suite [29]. These files were then converted into a pdbqt format using the built-in Autodock Vina feature in the Raccoon software [30].

#### 2.3.2. Pharmacophore-Based Library Generation

**Figure 2:**
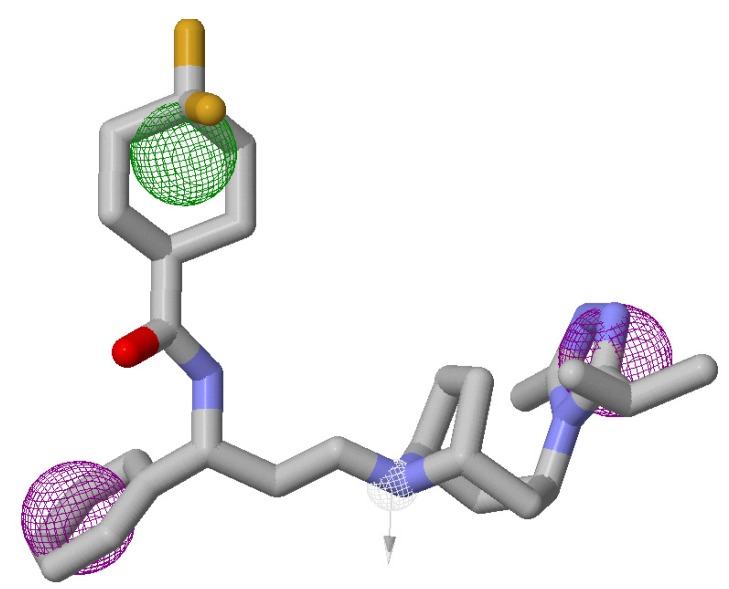
Maraviroc structure used as a template for pharmacophore-based and structural similarity-based compound library generation. Pharmacophore selection criteria—green depicts hydrophobicity, purple depicts aromatic and white depicts hydrogen donor. Arrows indicate that constraints have been imposed.

The pharmacophore-based compound libraries were generated using the chosen pharmacophoric regions as illustrated in Figure 2 and the ZincPharmer Database [31]. Also, keeping in mind that the desired compounds would be selected based on their probability of forming good interactions with the receptor. Furthermore, the Lipinski Rule of Five was imposed as the set criterion for screening compounds not only for confining conformational variations of the same ligand, but also for reducing any duplication The query search generated a total of 602 compound hits. All these compounds were downloaded as a single sdf file format and then separated and processed as explained above in Section 2.3.1.

### 2.4. Virtual Screening and Validation of Docking Protocol

In our study, the known CCR5 antagonist (maraviroc) and the two respective ligand-based compound libraries were subjected to virtual screening against the CCR5 enzyme. The Autodock Vina [32] screening software was used to conduct docking calculations. Although the Screening procedure was run using the software default settings; the exhaustiveness of the screening was fixed to the value of 8. The grid box used to define the screening site was verified by using the built-in functionality property found in Autodock Vina [24]. The grid box was defined around the following key amino acid residues, namely Phe85, Trp86, Trp 89, Leu104, Tyr108, Ile 198, Try251 and Glu283,and these resembled the active site residues found in the crystal structure of CXCR4 enzyme following the sequence alignment performed in Chimera. The X, Y and Z centres were defined as 11.01, −2.08 and 45.68, whereas the X, Y and Z size dimensions were defined as 58, 82 and 74, respectively. Autodock Vina screening results were produced in the pdbqt format. From each of the two compound libraries, the top ten compounds were selected on the basis of best binding affinities and visualized using the Viewdock feature in Chimera.

### 2.5. Molecular Dynamics Simulations and Post-Dynamic Analysis

The best-docked ligand-enzyme complexes that resulted from the structure-based and pharmacophore-based compounds library were then exposed to MD stimulations using the Amber software [33], following the procedure explained in our previous report [34] were performed using the We examined the post-dynamic nature of how ligands interacted with the CCR5 target protein within a range of 5 Å as illustrated by hydrogen bond and hydrophobic interactions using the Molecular Viewing Operator (MOE) program [35]. Likewise, residue contribution towards ligand binding was computed using the Moldock scoring functions [29].

### 2.6. Three-Dimensional (3D) QSAR Analysis

**Figure 3:**
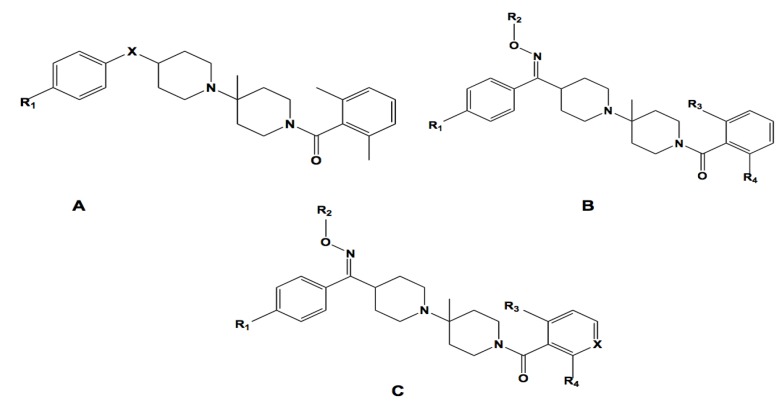
The 2D structures for the oxamino-piperidino-piperidine amide analogs used in the 3D QSAR of this work.

**Figure 4:**
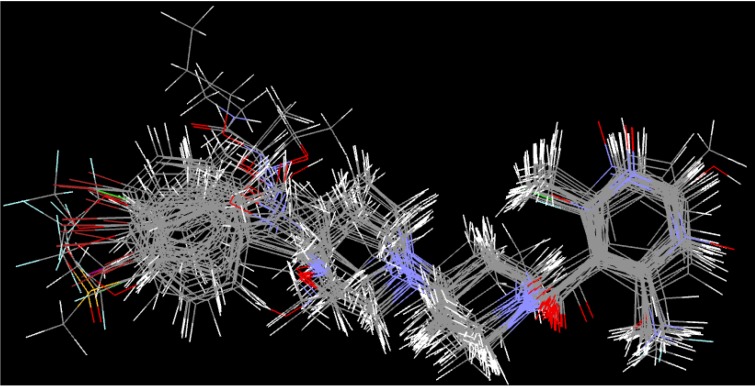
Molecular alignments used in the present study.

A set of 35 novel oxamino-piperidino-piperidine amide analogs (Figure 3) with available IC_50_ (mM) data was taken from literature for the development of the atom-based 3D-QSAR model (Table 1) [1]. This 3D-QSAR study was performed in Discovery studio 3.5 [36]. The 1/logIC_50_ value of CCR5 was used in this study. Of the 35 compounds reported, 26 compounds were used as a training set and the remaining nine compounds were used as a test set, based on a random selection. The compounds in the test set have a range of biological activity values similar to that of the training set. The ligands were pre-aligned using a molecular overlay method and placed in a 3D grid space (Figure 4). The grid spacing was 1 Å. The energy potentials on every grid point were then calculated using a CHARMm force field which used the electrostatic potential and the Van der Waals potential and treated as separate terms. A +1e point charge is used as the electrostatic potential probe and distance-dependent dielectric constant is used to mimic the solvation effect. For the Van der Waals potential a carbon atom with a 1.73 Å radius is used as a probe. The energy grid potentials can be used as independent variables to create partial least-squares (PLS). Furthermore, the best 3D-QSAR model was validated by predicting activities of the 9 test set compounds. The 3D-QSAR was evaluated by cross-validated R^2^, Q^2^. The predicted 1/logIC_50_ at 6th PLS factor are tabulated in Table 1.

**Table 1:** Dataset analyzed for 3D QSAR with experimental 1/logIC_50_, predicted 1/logIC_50_ and residual value.

#	Core	X	R_1_	R_2_	R_3_	R_4_	Expt. 1/logIC_50_ (mM)	Prdt. 1/logIC_50_ (mM)	Residual
1	A	CH_2_	Br	-	-	-	0.250	0.259	0.009
2	A	NH_2_	Br	-	-	-	0.267	0.265	0.002
3	A(E)	=N–OCH_3_	Br	-	-	-	0.290	0.270	0.020
4	A(E)	=N–OCH_3_	Br	-	-	-	0.252	0.271	−0.019
5	B	-	Br	CH_3_	Cl	NH_2_	0.360	0.359	0.001
6	B	-	Br	CH_3_	CH_3_	OH	0.278	0.276	0.001
7	C	N^+^–O	CH_3_	CH_3_	CH_3_	CH_3_	0.267	0.286	−0.018
8	C	N	Cl	C_2_H_5_	CH_3_	CH_3_	0.435	0.392	0.043
9	A	CH_2_	Cl	-	-	-	0.229	−0.248	−0.018
10	A	CH_2_	I	-	-	-	0.253	0.230	0.023
11	A	CH_2_	CF_3_	-	-	-	0.333	0.269	0.064
12	A	CH_2_	CH3	-	-	-	0.218	0.233	−0.015
13	A	CH_2_	OCH_3_	-	-	-	0.227	0.233	−0.006
14	A	CH_2_	SO_2_CH_3_	-	-	-	0.260	0.273	−0.013
15	A	C=CH_2_	Br	-	-	-	0.333	0.313	0.020
16	B(Z)	-	Br	H	CH_3_	CH_3_	0.250	0.245	0.005
17	B(Z)	-	Br	C_4_H_9_	CH_3_	CH_3_	0.266	0.275	−0.009
18	B	N	Br	CH_2_-CO-NHCH_3_	CH_3_	CH_3_	0.274	0.292	−0.016
19	B	N	Br	C_2_H_5_	F	CF_3_	0.301	0.305	−0.004
20	C	N^+^–O	Br	C_2_H_5_	H	CH_3_	0.318	0.289	0.290
21	C	N^+^–O	Br	C_2_H_5_	CH_3_	CH_3_	0.338	0.382	−0.044
22	C	N^+^–O	Br	C_2_H_5_	H	CH_3_	0.310	0.307	0.003
23	C	N^+^–O	CF_3_	CH_3_	CH_3_	CH_3_	0.329	0.324	0.005
24	C	N^+^–O	OCF_3_	CH_3_	CH_3_	CH_3_	0.270	0.298	−0.028
25	C	C=O	OCF_3_	C_2_H_5_	CH_3_	CH_3_	0.307	0.314	−0.007
26	C	-	Cl	C_2_H_5_	CH_3_	CH_3_	0.324	0.324	−0.006
27	A	N	Br	-	-	-	0.243	0.261	−0.018
28 ^t^	B	N^+^–O	Br	CH_3_	CH_3_	NH_2_	0.371	0.385	0.086
29 ^t^	C	CH_2_	Br	CH_3_	CH_3_	CH_3_	0.371	0.329	0.076
30 ^t^	C	CH–OH	Br	C_2_H_5_	CH_3_	CH_3_	0.301	0.302	0.001
31 ^t^	A	-	-	-	-	-	0.270	0.277	−0.007
32 ^t^	A	-	Br	-	-	-	0.205	0.254	−0.54
33 ^t^	B(Z)	N^+^ = O	Br	C_3_H_7_	CH_3_	CH_3_	0.310	0.271	0.38
34 ^t^	C		CH_3_	CH_3_	-	-	0.263	0.254	−0.091
35 ^t^	C		CF_3_	C_2_H_5_	CH_3_	CH_3_	0.321	0.306	0.015

*t* = test set. The conformation of compound denoted in brackets ( ) [1].

**Figure 5:**
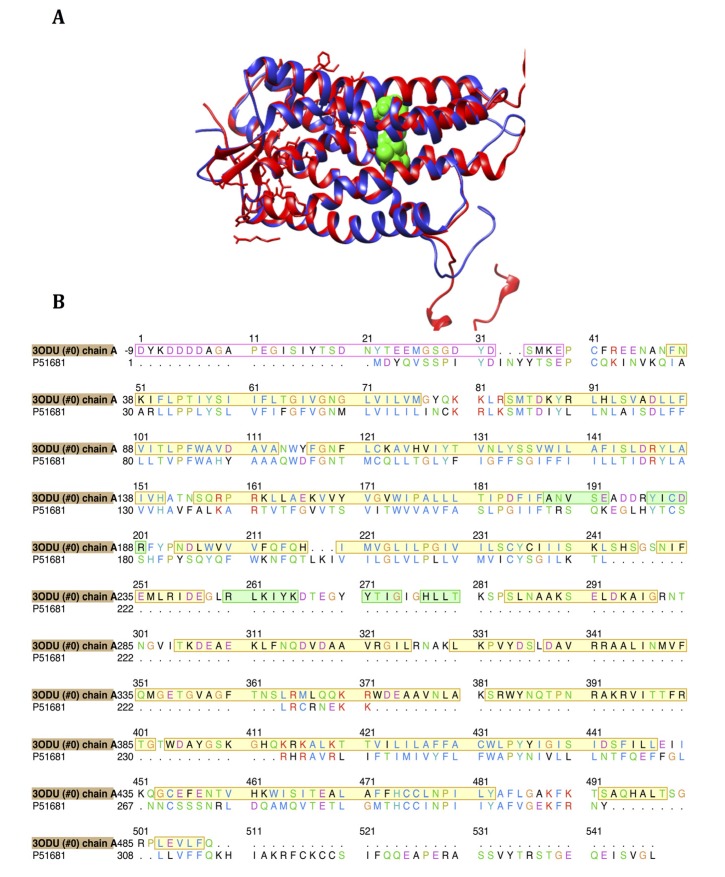
(**A**) Superimposed structures of 3ODU [16] and modeled CCR5 enzyme (blue) with CCR5 antagonist, maraviroc [24]; (**B**) The 2D sequence alignment of 3ODU and the homology model generated for our study. Yellow highlighting represents α-helices and green highlighting represents β-sheets. Sequences outlined in red lack 3D crystal structure.

## 3. Results and Discussion

### 3.1. Homology Modeling of CCR5

The actual homology model of human CCR5 was modeled using the 3ODU crystal structure as a structural modeling template. As outlined in Figure 5, both these proteins demonstrated good structural similarity in and around the active site residues, with most of the residues having relatively identical locations to each other. The Multi-align Viewer tool located in Chimera recorded a 42.11% shared similarity between the two proteins’ sequences; after modeling, the enzyme model had a zDOPE score of 0.91 with an RMSD of 1.1771 Å. Four differences were observed between the active site sequences of our modeled CCR5 and the 3ODU model template, which included Leu204 (3ODU) corresponding to Leu104 (CCR5), Trp85 corresponding to Trp86, and two residue gaps namely corresponding to Leu213 and Phe113 from 3ODU (Table 2), respectively. We assume that these noted differences have had very minimal effects in the docking study as result of the shared structural similarity between the Leu and Trp residues, respectively. Further investigation is required to verify this assumption. All non-modeled regions were removed from the active site in order to allow for emphasis on all crucial residues and their importance in the active site of CCR5.

**Table 2:** Comparison of the active site residues between the modeling template (3ODU) and modeled structure.

Active site residues (3ODU)	Corresponding modeled active site residues
Glu283	Glu283
Ile198	Ile198
Leu204	Leu104 #
Leu213	*
Phe85	Phe85
Phe109	Phe109
Phe113	*
Thr195	Thr195
Trp85	Trp86 #
Trp94	Trp94
Trp248	Trp248
Tyr89	Tyr89
Tyr108	Tyr108
Tyr251	Tyr251

# —Differing Residue; * —Residue Gap.

### 3.2. Virtual Screening

**Figure 6:**
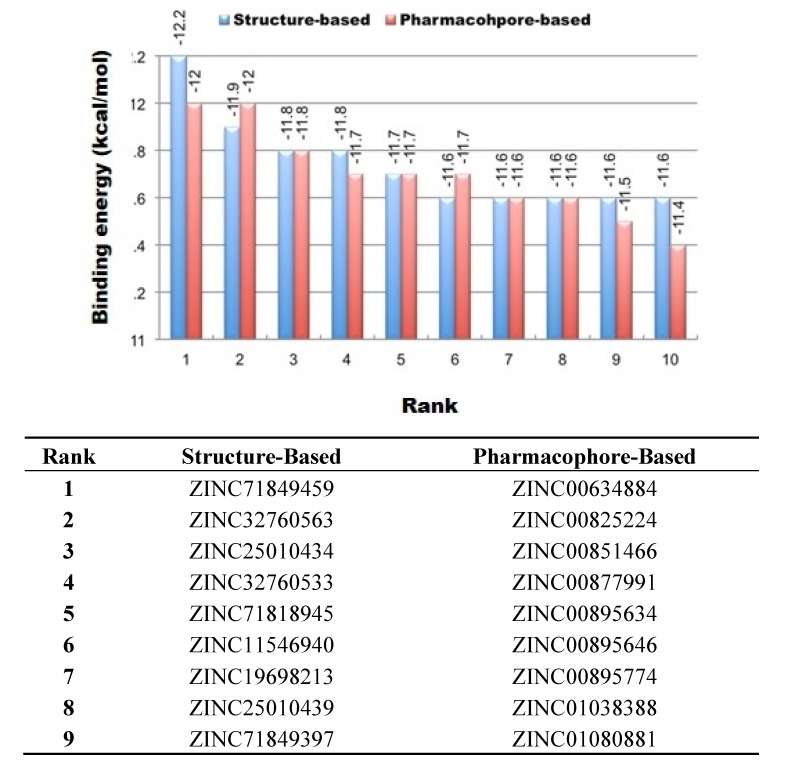
The top 10 ranked ZINC compounds from both the 2D similarity-based and pharmacophore-based libraries.

Results obtained from virtual screening for the pharmacophore-based and structure-based compound libraries are shown in Table 3. Our query of the Zinc Database for compounds bearing 2D shape similarity-based identity to the reference drug template generated 220 compound hits. However, our query of the ZincPharmer Database for pharmacologically related compounds generated 120 compound hits. All compounds in the two generated compounds libraries (see Methods sections for details) were then docked into the active site of the CCR5 enzyme using Autodock Vina and thereafter, from each library, we selected the top 10 compound hit leads. As shown in Table 3 and Figure 6, all top 10 ranked compounds from each library exhibited remarkably higher binding energies compared to Maraviroc (−10.2 kcal/mol), with binding energies ranging from −12.2 to −11.6 kcal/mol for the 2D shape similarity-based identity library, whereas the pharmacophore-based library had binding energies ranging from −12.0 to −11.4 kcal/mol.

As outlined in Figure 6, an unexpected observation revealed that compounds that were structurally similar had higher binding affinities on average compared to those compounds that were pharmacophorically-based. Although, not a huge variance amongst both libraries existed, however, this was an indicator about the particular importance of each specific pharmacophoric area required for CCR5 antagonistic behavior. This observation might be attributed to the selected pharmacophore-based groups. A further investigation is required for the imperative role played by these pharmacophoric groups in terms of site-specific interactions and in return, how do these interactions affect inhibitor binding affinities and functioning. It was also worth mentioning that compounds 3, 5, 7 and 8 from both the structure-based and pharmacophore-based generated libraries demonstrated exact binding energies (Figure 6). Another interesting observation, not a large difference existed amongst the top 10 binding energies compounds from both the structure-based and pharmacophore-based generated libraries with a difference of −0.60 kcal/mol between the highest and lowest ranked compounds (Figure 6). This observation might be attributed to the conservancy of the crucial pharmacophoric and architecturally shared properties “amongst these compounds allowing for “alleged stability”, and hence for well-maintained binding affinities.

**Figure 7:**
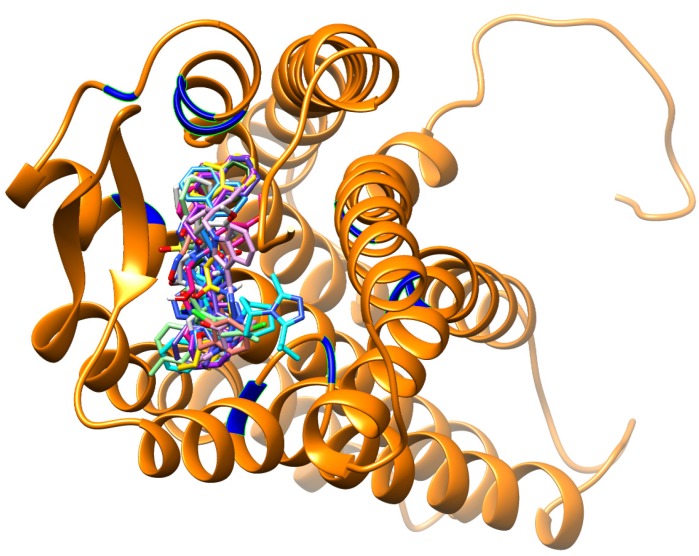
Docking conformations of the known CCR5 antagonist (maraviroc) and the top 10 ranked docked compounds from both the pharmacophore-based and structure-based libraries determined in this study, all-complexed with the CCR5 enzyme.

With the intention of finding the best compounds of this study, we integrated both the generated compound libraries and revealed the top 10 best-docked compounds (Figure 7 and Table 3). It was instantly observed that these compounds were remarkably larger in size as compared to maraviroc (Figure 7). Furthermore, majority of the compounds occupied the spaces between Tyr89, Trp94, Glu283, Leu104, Thr195, Tyr251, Phe109, Ile198 and Trp248 respectively, something which maraviroc did not achieve as a result of its smaller size (Figure 7). In this study, integration of these factors might be the contributing factors for the higher binding affinities as the total number of interactions with the active site was remarkably higher compared to those felt by maraviroc.

From our docking calculations, it appeared that the virtual screening compound hits demonstrated good activity as CCR5 antagonists. Several factors might have contributed to these findings, however one of such importance to us was that the docking protocol implemented might have not been accurate enough to provide precise estimates of the different binding energies. In an effort to eradicate this factor, we opted to validate the docking method applied in this study. “Cross validation” was not employed to validate our docking results. This is an approach, where other docking programs are used to validate the data attained from the original docking software. Due to previous experience of using different docking software with various scoring functions would generate results that could be different and be misleading, we opted to disregard this docking validation approach. We strongly believe that the utmost rational mannerism for validation of docking calculations, or even any other computational tool, is to perform the calculations on a set of compounds with available experimental data and these results are then compare against known experimental data for validation.

**Table 3:** List of the top 10 screened compounds based on their docked binding energy. Compounds are ranked in order of highest to lowest binding affinity.

Library	Rank	ZINC ID	Structure	Binding Energy (kcal/mol)	xlogP	H-bond Donors	H-bond Acceptors	Molecular Weight (g/mol)
**Ref**	**R**	ZINC03817234	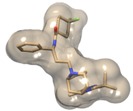	−10.2	−3.50	2	6	514.69
S *	**1**	ZINC71849549	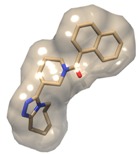	−12.2	2.27	2	6	318.89
P **	**2**	ZINC00825224	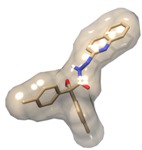	−12.0	4.11	3	5	397.488
**P**	**3**	ZINC00634884	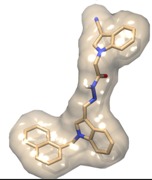	−12.0	5.96	1	6	481.60
**S**	**4**	ZINC32760563	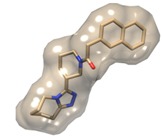	−11.9	3.47	0	5	388.51
**S**	**5**	ZINC32760533	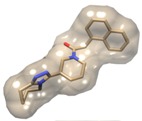	−11.8	3.44	0	5	388.52
**S**	**6**	ZINC25010434	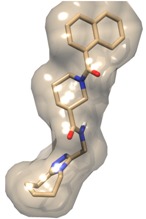	−11.8	2.16	1	7	431.54
**P**	**7**	ZINC00851466	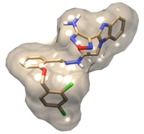	−11.8	5.52	3	10	536.38
**S**	**8**	ZINC71818945	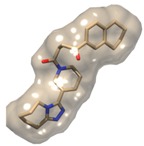	−11.7	3.35	0	6	434.58
**P**	**9**	ZINC00895646	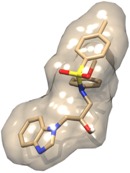	−11.7	3.99	2	7	451.55
**P**	**10**	ZINC00895634	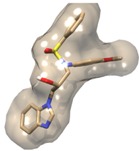	−11.7	3.54	2	7	438.53

* Similarity-based library, ** Pharmacophore-based library, Ref-Maraviroc.

**Figure 8:**
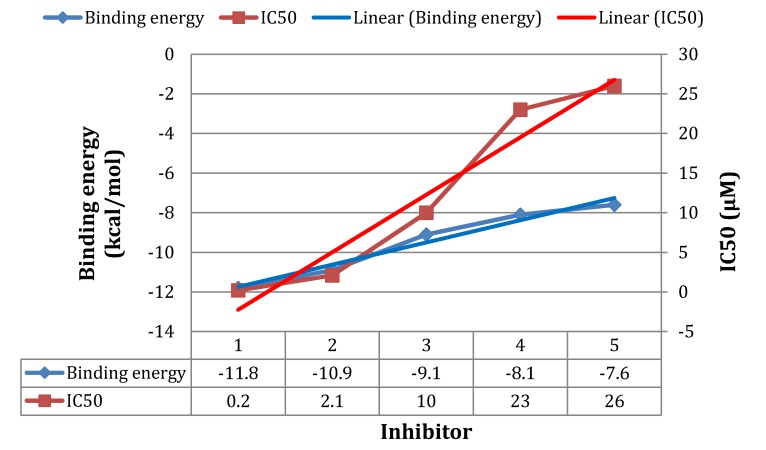
The binding energies determined in our study and were compared against IC_50 _values for the compounds assayed. The higher the binding affinity, the higher the IC_50_.

To this end, in order to validate our docking approach implemented in this study, we performed docking analyses on a set of compounds assayed using our docking method. The structures and experimental IC_50_ values were obtained from Binding database [37]. The binding energy of each compound was then plotted against its corresponding experimental IC_50_ value (Figure 8). As evident from the docking results (Figure 8), the docked energies are in great accordance with the experimental IC_50_. We observed that as the binding affinity increased (lower binding energy), the IC_50_ increased (Figure 8). We postulate that the larger the binding affinity, the more concentration is needed for complete enzyme inhibition. To us, this proves to be an interesting trend, since the top 10 ranked compounds which were obtained from both libraries (Table 3) had higher binding energies compared to any of those used in the assay. This implies that the docking approaches used in this work could be reliable enough to estimate the binding affinities for the top 10 ranked compounds (from both libraries—Table 3).

### 3.3. Molecular Dynamics Simulations

In order to gain more insight into the stability of the resulted highest ranked virtual screening hit complexes, the nature of overall interaction theme between the proposed ligands and the target protein and the specific amino acids involved in ligand binding, we performed 1 ns MD stimulations followed by extensive post-dynamic analyses on the ligand-enzyme complexes.

**Figure 9:**
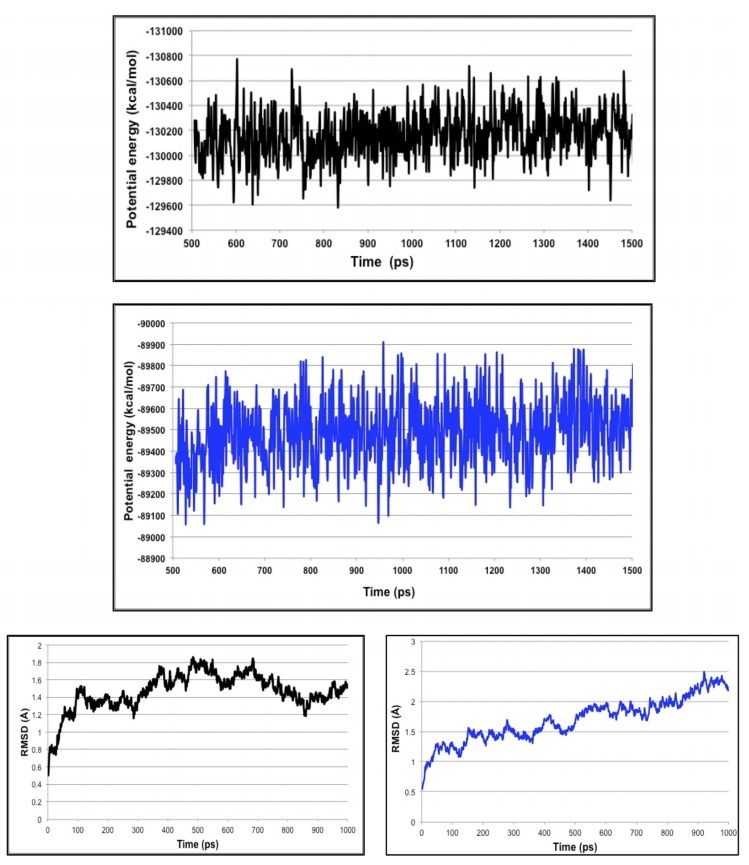
The highest ranked virtual screening hit lead complexes with CCR5 subjected to MD simulations. Structure-based compound (ZINC71849459) in complex with CCR5 (Black). Pharmacophore-based compound (ZINC00634884) with CCR5 (Blue).

MD stimulations of 1 ns were performed for the highest ranked virtual screening hit complexes (Figure 9), to ensure the stability of the ligand within the CCR5 active site. We also performed MD simulations on the reference ligand (maraviroc) bound to CCR5 (see Supplementary Material). From our previous experience with molecular docking, in many occasions, we experienced that even best docked structures may fly away from the enzyme active site within a few picoseconds of MD stimulations. Therefore, we believe that docking calculations that are not validated by relatively a long MD run to ensure stability of the system might not be reliable. Interestingly, for all the compound-enzyme complexes, the average RMSD values were below 2.5 Å. In addition, the variability of the potential energies fell with 1000 kcal/mol and this suggested being a good indicator of the system stability.

### 3.4. Per-Residue Interactions

**Figure 10:**
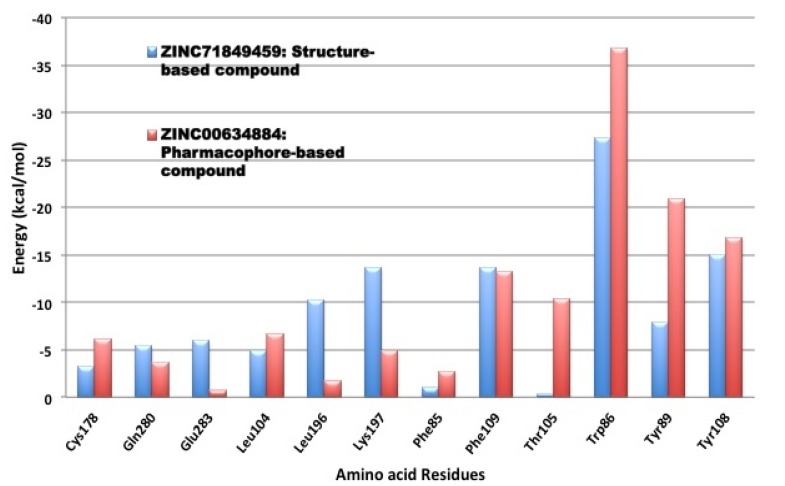
Per-residue interactions for the highest ranked compounds with the best binding energy from the structure-based and pharmacophore-based libraries.

**Figure 11:**
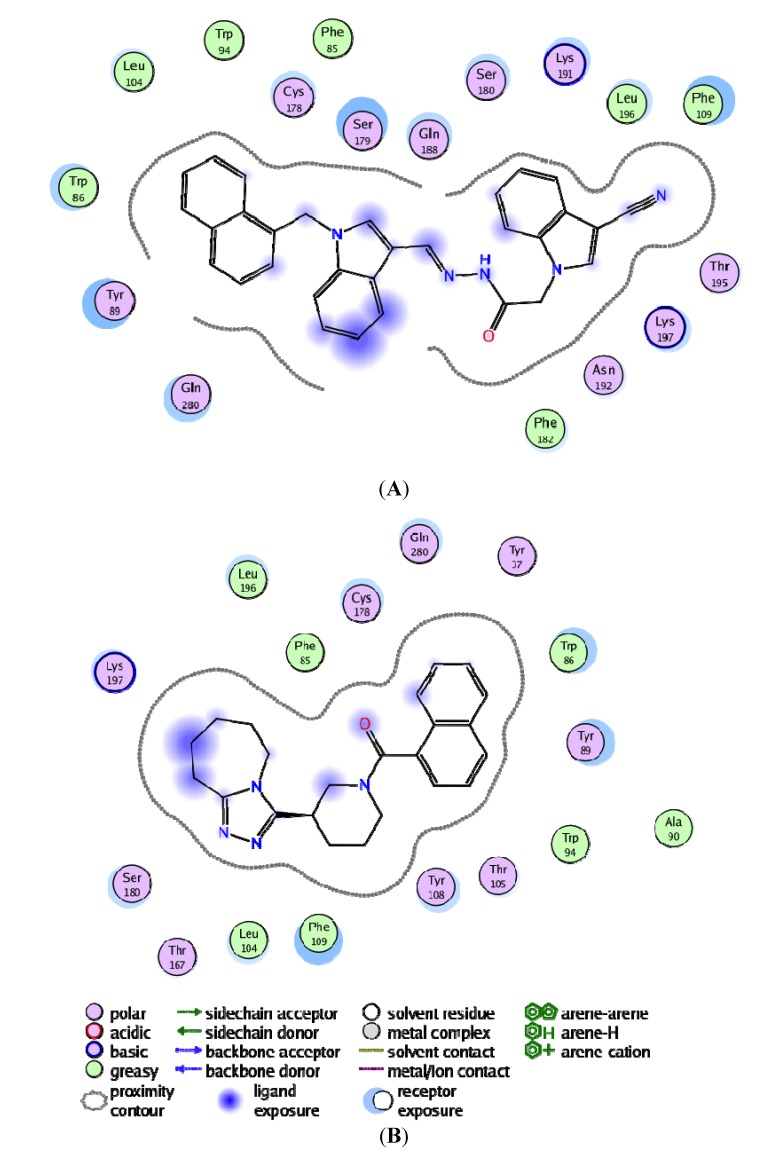
Pharmacophore-based compound (ZINC00634884) with CCR5 (**A**) and Structure-based compound (ZINC71849459) in complex with CCR5 (**B**), respectively, showing the hydrogen bonding and electrostatic interactions with the enzyme’s active site using MOE.

In an effort to investigate the contribution of a single amino acid towards ligand (and/or antagonist) binding, we computed per-residue interactions using Moldock software [29] (Figure 10). The top ranked antagonist with the highest binding energy within each compound library was assessed, which included ZINC71849459 from the structure-based identity library and ZINC00634884 from the pharmacophore-based library. We noticed that Lys197, Phe109, Trp86, Tyr89 and Try108 exhibited remarkably interactions with both of those docked ligands. However, Trp86, Tyr89, Thr105, and Tyr108 demonstrated especially better interactions with ZINC00634884 (pharmacophore-based library), whereas Lys197, Leu196 and Glu283 showed good interactions with ZINC71849459 (structure-based identity library). As shown in Figure 11, in order to gain a better understanding of the ligand-amino acid interactions occurring within CCR5’s active site, we generated a plot of the specific amino acid residues-ligand interactions using the MOE software [35].

The MOE plot analysis of ZINC71849459 bound to CCR5 enzyme’s active site revealed that the ligand was especially well surrounded electrostatically by several amino acid residues within the active site (Figure 11A). However, it could be noted that the ligand ZINC00634884 bound to the active site was not well cradled electrostatically by the amino acid residues compared to the former (Figure 11B). An interesting observation was that not a single amino acid formed any hydrogen bonds between the protein and ligand for anyone of the two ligands investigated. As shown in Figure 11, on average the hydrophobic amino acid residues played crucial roles in protein-ligand interactions than the hydrophilic amino acid residues. Another important observation was that the stronger ligand-amino acid residue interactions exhibited here in Figure 11 matched to those outlined in Figure 10.

### 3.5. Atom-Based 3D-QSAR

**Figure 12:**
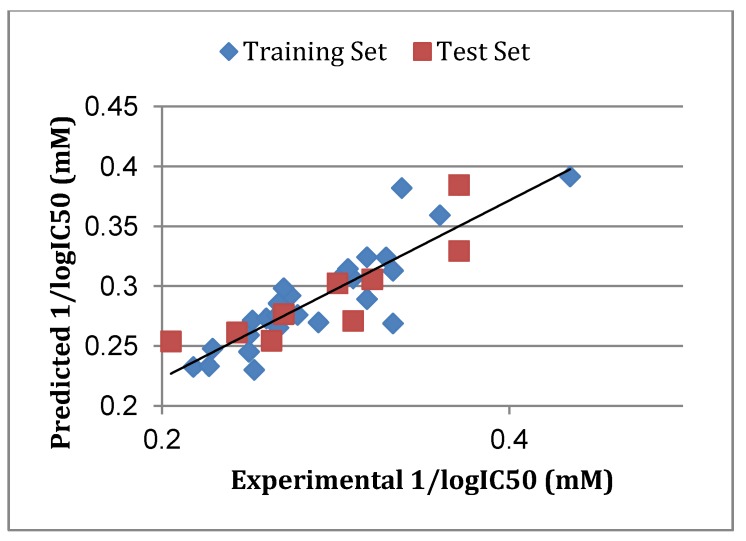
Correlation graph between the experimental 1/log IC50 and predicted 1/logIC50.

The atom-based 3D-QSAR model was developed from the training set of 26 inhibitors (Table 1) and the test set of 9 inhibitors using molecular overlay alignments (Figure 3). This atom-based 3D-QSAR model was built after model development and validation based on the internal predictions of the training set and the external predictions of the test set. PLS analyses of the CCR5 inhibitor training sets showed a high cross-validated *r*_cv_^2^ value of 0.84 using three principal components and non-cross-validated *r*^2^ value of 0.941. All of the parameters of these QSAR model showed certain reliability and feasible predictability to help us design new and high selectivity CCR5 inhibitors. From Figure 12, we can see that almost all compounds in the test set and training set yielded a good predicted value. The graphical plot of observed *vs.* calculated TPH1 inhibitory activity for both the training set as well as the test set is shown in Figure 12.

## 4. Conclusions

In the present work, the structure of human CCR5 was homology modeled using the crystal structure of CXCR4 as a structural modeling template. The protein appeared to be modeled with a remarkably degree of accuracy, specifically at the active site where docking studies were performed. Our query of the ZINC database for drug-like compounds that shared 2D shape similarity-based identity and query of ZINCPharmer for pharmacologically related drug-like compounds to a known FDA-approved CCR5 antagonist called maraviroc. The entire top 10 ranked compounds from each library exhibited remarkably higher binding energies compared to the best-docked structure of maraviroc. Moreover, an unforeseen observation revealed that compounds that were architecturally similar had higher binding energies on average compared to those that were pharmacophorically-based. To validate our docking calculations, the same docking approach adopted for the ligand-based libraries was performed on a set of compounds with known experimental data attained from inhibition assays against HIV-1 CCR5 and the results were compared against experimental data. The docked energies are in great accordance with the experimental IC_50_. It was concluded that compounds with more favorable predicted binding energies than the known drug maraviroc will have “better activity” (*i.e.*, smaller IC_50_ values). To us, this proves to be an interesting trend, since the top 10 ranked compounds which were obtained from both libraries had higher binding energies compared to any of those used in the biological assay, therefore it stands to reason compound hits elucidated in this study exhibit humble activity as CCR5 antagonists. Also, this implies that the docking approaches used in this work could be reliable enough to estimate the binding affinities for other compounds to be studied. Furthermore, from our previous experience with molecular docking, and in numerous insistences, the reliability of a stable protein-ligand complex might not be a true reflection. Therefore, in order to obtain more insight on the stability of the resulted docked complexes, the nature of the overall interaction themes between the generated ligands and the target protein, and the specific amino acids involved in the ligand binding, we performed 5 ns MD simulations followed by extensive post-dynamic analyses on the ligand-enzyme complexes resulted from our docking simulations. We took our study a step further by obtaining a set of 35 novel oxamino-piperidino-piperidine amide analogs with available IC_50_ (mM) data taken from literature for the development of our atom-based 3D-QSAR model. All of the parameters of the QSAR model showed certain reliability and feasible predictability to help us design new and high selectivity CCR5 inhibitors. Our novel identified leads have the propensity to be considered as potential CCR5 antagonists and moreover as potential HIV-1 entry inhibitors. Information gained from this study could shed light on the activity of a new series of lead compounds as potential HIV entry inhibitors and should serve as a powerful tool in the drug design and development machinery.

## Supplementary Materials

Supplementary materials can be accessed at: http://www.mdpi.com/1420-3049/19/4/5243/s1.
