# Supplementary material for: Integrated Computational Tools for Identification of CCR5 Antagonists as Potential HIV-1 Entry Inhibitors: Homology Modeling, Virtual Screening, Molecular Dynamics Simulations and 3D QSAR Analysis
Source: Molecules. 2014 Apr 23;19(4):5243–65. doi: 10.3390/molecules19045243 (PMC6270745; doi:10.3390/molecules19045243)

Supplementary Information

Figure S1. The reference drug (Maraviroc) bound to CCR5 was subjected to 1 ns MD simulations.

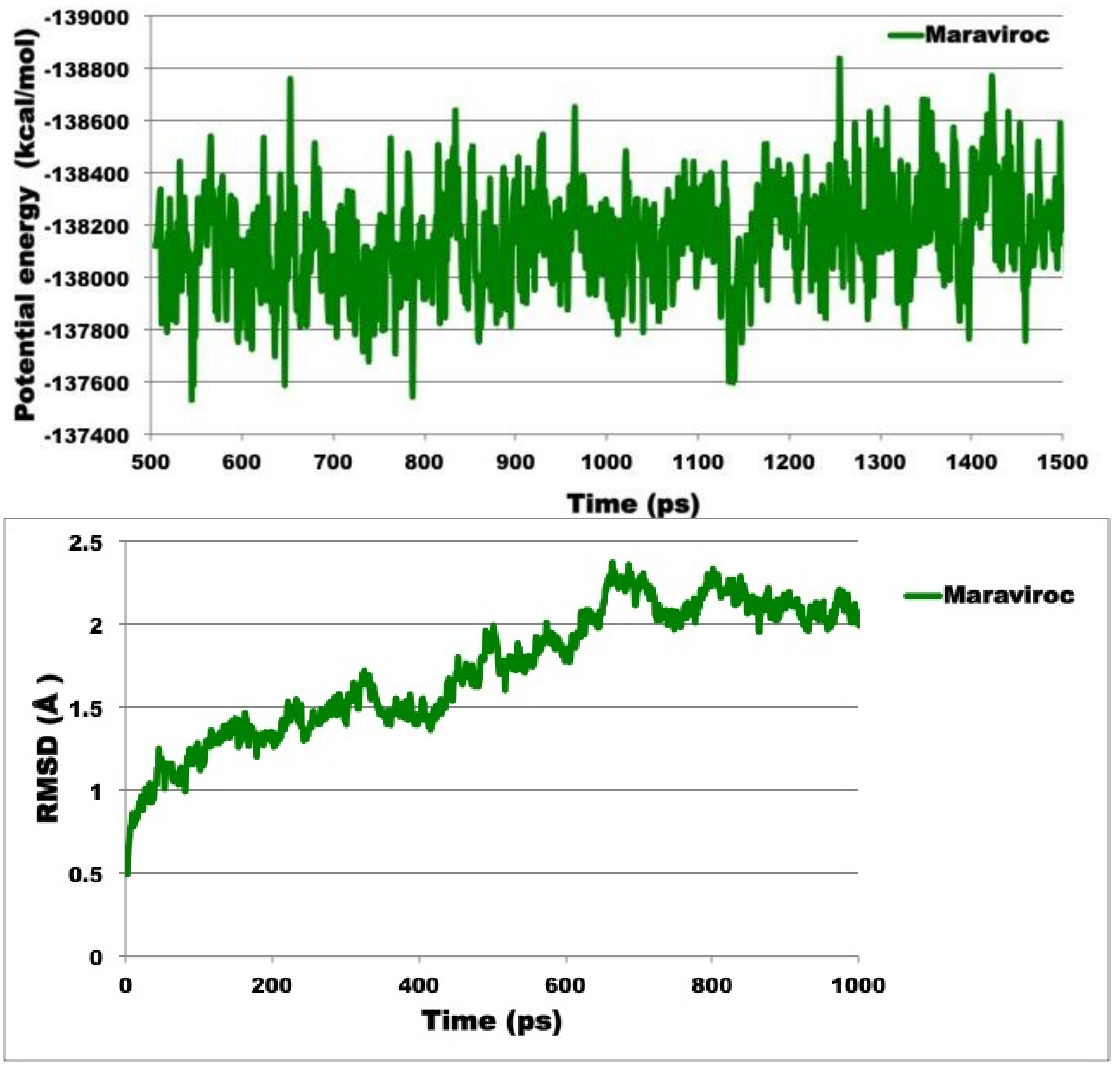

Supplement: Supplementary file 1 [file molecules-19-05243-s001.pdf]
